# Association between Circadian Clock Genes and Diapause Incidence in *Drosophila triauraria*

**DOI:** 10.1371/journal.pone.0027493

**Published:** 2011-12-02

**Authors:** Hirokazu Yamada, Masa-Toshi Yamamoto

**Affiliations:** Drosophila Genetic Resource Center, Kyoto Institute of Technology, Kyoto, Japan; Pennsylvania State University, United States of America

## Abstract

Diapause is an adaptive response triggered by seasonal photoperiodicity to overcome unfavorable seasons. The photoperiodic clock is a system that controls seasonal physiological processes, but our knowledge about its physiological mechanisms and genetic architecture remains incomplete. The circadian clock is another system that controls daily rhythmic physiological phenomena. It has been argued that there is a connection between the two clocks. To examine the genetic connection between them, we analyzed the associations of five circadian clock genes (*period*, *timeless*, *Clock*, *cycle* and *cryptochrome*) with the occurrence of diapause in *Drosophila triauraria*, which shows a robust reproductive diapause with clear photoperiodicity. Non-diapause strains found in low latitudes were compared in genetic crosses with the diapause strain, in which the diapause trait is clearly dominant. Single nucleotide polymorphism and deletion analyses of the five circadian clock genes in backcross progeny revealed that allelic differences in *timeless* and *cryptochrome* between the strains were additively associated with the differences in the incidence of diapause. This suggests that there is a molecular link between certain circadian clock genes and the occurrence of diapause.

## Introduction

Seasonal photoperiodic responses are observed in many organisms, including plants, fungi, birds, mammals, and arthropods [1]–[3]. Diapause is a photoperiodic response that often results in a delay in a specific developmental stage; it is broadly observed in insects and other arthropods and is considered to be an adaptation to unfavorable seasons or conditions.

Diapause often depends on day (or night) length and temperature and is observed over a wide range of taxa. However, the physiological processes and genetic architectures involved in diapause are still largely unknown [4]. In contrast, the circadian clock, which controls daily rhythmic physiological phenomena, including locomotor activity, eclosion, and oviposition [5]–[7], has been studied well at the molecular level [8]–[10]. Both types of biological clock involve time-dependent responses to light stimulation [11]. Since Bünning [12] first proposed a two-clock connection whereby the daily circadian clock forms the basis of the seasonal photoperiodic timer, there has been a long argument—over several decades—about whether insect photoperiodism follows this theory [10], [11]. Many studies, performed in various species, have resulted in several models for explaining photoperiodic function [13]–[19]. One of the most widely adopted strategies for testing the involvement of circadian clock oscillation in the photoperiodic clock is the Nanda-Hamner protocol [20]. Although this protocol often produces positive results [14], [21], [22], it has also failed under different conditions, or even with different strains within the same species [4], [19], [23]. An alternative, nonoscillatory, hourglass-like model has also been proposed [15], [19]. This model was successfully applied by Veerman and Vaz Nunes in a spider mite [18]. However, the species of spider mite used also demonstrated a strongly positive Nanda-Hamner effect, suggesting that the photoperiodic clock was based on circadian clock oscillation [24]. Similar inconsistent results from the two models have been obtained in other insects [25]–[27]. Thus, the extent—if any—of dependence of the photoperiodic clock on the circadian clock remains obscure.

To further define the involvement of circadian clocks with photoperiodic responses, we performed a genetic analysis of the two clocks to determine whether common genes were present in the two clock systems. We chose the fruit fly *Drosophila triauraria*, a close relative of *Drosophila melanogaster*, for our analysis [28]. Circadian clock mechanisms have been well studied in *D. melanogaster*, but photoperiodicity in this insect's reproductive diapause is difficult to detect [29]. In contrast, *D. triauraria* has a distinct reproductive diapause with a robust photoperiodic response. Ovarian development in *D. triauraria* occurs normally with long daylength at moderate temperatures (e.g. 15°C) but not under short daylength conditions even at the same temperature [30], [31]. Geographic variation in diapause intensity is known to occur, and non-diapause populations (i.e., those that do not enter diapause, even under short daylength conditions) have been described [30], [31].

Circadian clocks consist of feedback loops involving several genes that are well conserved across broad taxa, from fungi to mammals [32], [33]. In *D. melanogaster,* five major genes are known in the central components of the circadian clock: *period* (*per*), *timeless* (*tim*), *Clock* (*Clk*), *cycle* (*cyc*), and *cryptochrome* (*cry*) [10]. Two intracellular feedback loops function in the circadian clock oscillators encoded by four of these genes, namely *per, tim, Clk*, and *cyc*. These gene products function as PER/TIM and CLK/CYC heterodimers. The CLK/CYC heterodimer activates the transcription of *per* and *tim*, while simultaneously repressing the transcription of *Clk*. Likewise, PER/TIM heterodimers repress their own production. The CRY protein encoded by *cry* is photosensitive and mediates entrainment of the clock by light [34], [35]. At the onset of photophase, CRY stimulated by light binds to TIM and then promotes degradation of TIM by phosphorylation [35]. These five genes are therefore central components of the circadian clock and were the focus of our study.

Although *D. triauraria* has tested positive for the involvement of circadian clock oscillation in the photoperiodic clock by using the Nanda-Hamner protocol [36], clear genetic covariance has not been detected between circadian-based oscillation resonance and diapause incidence [37]. Alternatively, an hourglass timer has been considered, assuming that diapause-inducing substances accumulate gradually, late at night [36]–[38]. This assumption of an hourglass timer and the lack of genetic covariance between circadian-based oscillation resonance and diapause incidence do not necessarily deny the involvement of circadian clock genes in diapause. Indeed, the involvement of a circadian clock has been suggested as part of photoperiodic time measurement in this species [36], [39]. Furthermore, recent studies suggest non-clock functions of circadian clock genes in the diapause occurrence [40], [41].

Here, we describe distinct phenotypes between diapause and non-diapause strains of *D. triauraria* and analyze the involvement of circadian clock genes in the photoperiodic response. Our results clearly demonstrate a correlation between diapause and two genetic loci that include *tim* and *cry*, thus providing a crucial link to the occurrence of diapause in *D. triauraria*.

## Methods

### Flies

We used four strains of *D. triauraria*, ONM, OEB12, KMJ1, and KMJD2. All the strains were established from natural populations in Japan. ONM was obtained from Onuma (42.0°N, 140.4°E) in 1981 (this strain was kindly provided by SG Goto). Strain OEB12 was collected on Okinoerabujima Island (27.2°N, 128.3°E) in 2005. Strains KMJ1 and KMJD2 came from Kumejima Island (26.2°N, 126.5°E) in 2002 and 2003, respectively. Strains OEB12, KMJ1, and KMJD2 were provided from the Drosophila Stocks of Ehime University (http://kyotofly.kit.jp/cgi-bin/ehime/index.cgi). All strains were maintained in glass vials containing standard cornmeal–yeast–glucose–agar medium at 23°C and a daily cycle of 15 h light:9 h dark.

### Assay for ovarian diapause

We conducted a photoperiodic response assay under two light-cycle conditions, 15 h light:9 h dark (long daylength, LD) and 10 h light:14 h dark (short daylength, SD). These conditions were chosen because the critical daylength of ONM (the diapause strain) was 13 h [37].

Flies were allowed to lay eggs for 1 day at 23°C under the LD light cycle, and then the food vials with laid eggs were moved to either LD or SD conditions at 15°C. Virgin females were collected within 8 h after eclosion and placed into new food vials under the same conditions. Sixteen days after eclosion, the flies were dissected in PBS and ovary development was examined. Females containing only oocytes that did not develop beyond stage 7 were defined as being in diapause [42], [43]. This assay was conducted on all four original strains described above and on hybrid F_1_ females from crosses between ONM (the diapause strain) and the other three strains (non-diapause strains). We also examined the progeny from reciprocal backcrosses of strains ONM and OEB12.

### DNA extraction

Genomic DNA of the two strains ONM and OEB12 was extracted individually with a DNeasy 96 Tissue kit (QIAGEN, Valencia, CA) in accordance with the protocol for animal tissues in the QIAGEN manual. DNA was eluted in 200 µl of buffer AE, of which 1 µl was used for amplification by PCR.

### Strain-specific molecular markers of circadian rhythm genes

To identify strain-specific molecular markers for the five circadian rhythm genes (*per*, *tim*, *Clk, cyc,* and *cry*), we amplified several regions within each of the genes by using PCR using ExTaq polymerase (TaKaRa, Shiga, Japan). Primers for the PCR and sequencing for genotyping are shown in Table 1. Amplified PCR products were purified with an ExoSAP-IT PCR-cleanup kit (USB, Cleveland, OH) and then sequenced with an Applied Biosystems (Foster City, CA) 3130*xl* Genetic Analyzer. Sequencing reactions were conducted with a BigDye Terminator v1.1/v3.1 Cycle Sequencing Kit (Applied Biosystems). Purification with ExoSAP-IT and cycle sequencing reactions with the BigDye Terminator were performed according to the manufacturer's instructions.

**Table 1:** Primers used for PCR and sequencing.

Gene		Primer sequences	Annealing temp. for PCR
*per*	F	5′-TTCTGCTGCGTCATCTCCATGC-3′	51°C
	R	5′-GGAACTCTTACTGTCATAGTAGTC-3′	
	S	5′-GACTTTGTGCACATCAAGGA-3′	
*tim*	F	5′-TGATACCVYTGCTGGAGAATGCC-3′	63°C
	R	5′-TNGTGTCDATGTGCTCCATRTCC-3′	
	S	5′-GAACCAGGAGTCCATCTCCA-3′	
*Clk*	F	5′-TGGTCAARTTYGTKGGYTACTTTC-3′	55°C
	R	5′-SGCATAGCTGACMACYTTRTGRGTG-3′	
	S	5′-GAAATGAGCATYATYGATCCSAC-3′	
*cyc*	F	5′-CAAAAYCACAGYGAGATCGAGAARCG-3′	53°C
	R	5′-ATRTAGTCAATYTCGYTYGTCC-3′	
	S	5′-TGAGCTCTCCTCCATGATCC-3′	
*cry*	F	5′-AGCGAATGTGATCTGGTTCC-3′	50°C
	R	5′-ATGCTCAGGCAGATCTCGTT-3′	
	S	5′-ACACAGGCTCGCAACTGGAC-3′	

F, forward primer; R, reverse primer; S, sequence primer.

### Generation of backcross progeny, and phenotyping and genotyping

We used two strains, the diapause strain ONM and the non-diapause strain OEB12, to generate backcross progeny (BC). First, to obtain F_1_ females, we performed two reciprocal crosses, ONM females ×OEB12 males, and OEB12 females × ONM males. We refer to the F_1_ flies from the former cross as F_1_(ONM×OEB12) and those from the latter cross as F_1_(OEB12×ONM). Then, BC females were produced by backcrossing both of the reciprocal F_1_ females to the OEB12 males. All of these crosses were performed under LD conditions at 23°C, and the mated F_1_ females were allowed to lay eggs under the same conditions for 1 day in a food vial. The food vials with laid eggs were then moved to two different conditions: LD and SD at 15°C. Ovarian development was inspected for diapause on day 16 post-eclosion, as described above. Dissected females were kept at −20°C for DNA extraction and genotyping. Genotyping was conducted for the five genes by examining strain-specific molecular markers with sequencing after PCR amplification. We used the following conditions for PCR to genotype the genes: 1.2 µl 10× ExTaq buffer, 0.96 dNTPs, 3.0 µl of 2-µM forward primer, 3.0 µl of 2-µM reverse primer, 0.15 µl ExTaq polymerase, 1.0 µl DNA template, and 2.69 µl distilled water for a final reaction volume of 12 µl. Reaction conditions were 94°C for 2 min plus 40 cycles of 94°C for 30 s, 50 to 63°C annealing for 30 s (51°C for *per*, 63°C for *tim*, 55°C for *Clk*, 53°C for *cyc*, 50°C for *cry*), 72°C for 1 min 30 s, plus a final 72°C extension for 7 min.

### Eclosion rhythm

Circadian rhythms were measured by using the pattern of adult eclosion under 12 h light:12 h dark at 23°C. In our preliminary experiments, we found only white flies (indicating that they had just eclosed) at the onset of the photoperiod. We therefore counted eclosed flies every hour during the 12 h of photophase. The white flies found at the onset of photophase were counted as those eclosed during the 1 h before the onset of photophase. The degree of rhythmicity in eclosion was measured by using Winfree's R-values [44]. The R-values were calculated on the basis of summation of the diel distributions of eclosion. First, the 8-h period of the day containing the highest number of eclosions was identified. The R-value was then calculated by dividing the number of eclosions within the remaining 16 h (i.e., outside the 8-h period) by the number within the highest 8 h and multiplying by 100. R-values of less than 30 were considered to be “highly rhythmic,” whereas those over 90 were “arrhythmic” [45].

### Statistics

We applied the generalized linear model assuming a binomial distribution of diapause occurrence (diapause or not) to analyze the effects of allelic differences in the clock genes on diapause incidence. Statistical calculations for the generalized linear model were performed by using the “glm” function in the “stats” package of the R statistical software package version 2.8.0 ([46], http://www.r-project.org/). The best-fit model was determined by using stepwise model selection with the Akaike information criterion (AIC) contained within the “MASS” package in R, choosing the AIC with the least score as the best fit. In the model selection, we included not only the five genes as main factors but also all possible interactions among the five genes.

## Results

### Diapause incidence

**Figure 1:**
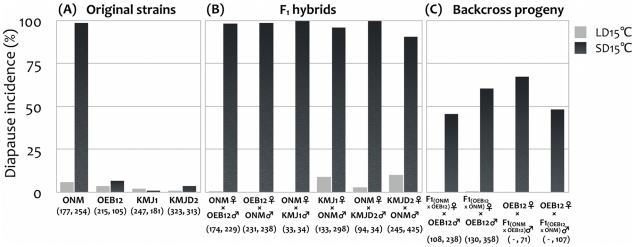
Diapause incidences in the original strains (A), F_1_ hybrids (B), and BC progeny (C) of *Drosophila triauraria*. LD, long daylength conditions (15 h light:9 h dark); SD, short daylength conditions (10 h light:14 h dark). Under both conditions the temperature was 15°C from egg to adult. Diapause incidences under LD are shown as gray bars, and those under SD are shown as black bars. Crosses from which F_1_ and BC females were obtained are shown under each bar. F_1_(ONM×OEB12) and F_1_(OEB12×ONM) indicate F_1_ flies obtained from ONM♀×OEB12♂ and OEB12♀×ONM♂, respectively. Numbers of dissected females are shown in parentheses: within the parentheses the values at left are those under LD conditions and those at right are under SD conditions.

**Figure 2:**
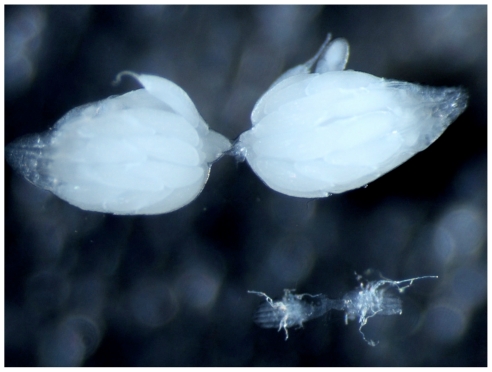
Pairs of ovaries dissected from 16-day-old female *Drosophila triauraria* reared under short daylength conditions at 15°C. Top: non-diapause strain (OEB12). Bottom: diapause strain (ONM).

We defined diapause as occurring in those 16-day-old females that contained only oocytes that did not develop beyond stage 7 (i.e., no yolk accumulation in the egg chambers). Using this criterion, no, or very little, diapause was observed in the four strains, or in the hybrid F_1_ and BC females, when they were reared under LD 15°C conditions (Fig. 1). However, striking differences were observed among females reared under SD 15°C conditions (Figs. 1 and 2): the high-latitude strain ONM displayed a very substantial diapause incidence (98.8%), whereas the low-latitude strains OEB12, KMJ1, and KMJD2 exhibited very low diapause incidences (6.7%, 1.1%, and 3.8%, respectively) (Fig. 1A). All females that were F_1_ hybrids between the diapause strain ONM and the non-diapause strains displayed high diapause incidences under SD 15°C conditions (90.8% to 100%, Fig. 1B); these incidences were similar to those observed in the diapause strain ONM. These results strongly indicate that the diapause strain had a dominant genetic effect on the phenotypes of heterozygous females.

### Circadian clock genes of *D. triauraria* and strain-specific molecular markers

We used the PCR primers shown in Table 1 to amplify genomic regions of the circadian clock genes *per*, *tim*, *Clk*, *cyc*, and *cry*. The PCR products were sequenced and BLAST searches confirmed their high degrees of similarity to the corresponding genes in *D. melanogaster*, as follows: *per* 86.8% (738/850 bp), *tim* 85.7% (275/321 bp), *Clk* 89.3% (134/150 bp), *cyc* 87.6% (686/783 bp), and *cry* 81.4% (544/668 bp). These regions are all exonic regions in *D. melanogaster*.

We identified at least one molecular marker—either a single nucleotide polymorphism (SNP) or insertion/deletion within each gene—that was useful for distinguishing allelic differences between the diapause strain ONM and the non-diapause strain OEB12 (Table 2): two strain-specific SNPs in *per*, an ONM-specific 12-base deletion and one strain-specific SNP in *tim,* one strain-specific SNP in *Clk*, three strain-specific SNPs in *cyc*, and one strain-specific SNP and an OEB12–specific 12-base deletion in *cry*. For each gene, we confirmed the strain specificity of these markers in more than 30 flies of each strain. We also confirmed the heterozygosity in the locations of the sequences from F_1_ flies between the strains. Furthermore, we found no contradictions in the genotyping results in 329 BC flies. These strain-specific markers were found in either coding or noncoding regions of sequences corresponding to those of *D. melanogaster* in the alignment results, as shown in Table 2 (coding regions are shown as boldface characters). These strain-specific markers were used for genotypic analysis of diapause in BC females.

**Table 2:** Strain-specific SNPs/deletions in the five clock genes in *Drosophila triauraria.*

*per*	ONM	**GAAGAGCACCTTCTGCGTGATGT(180bp)GCAGCTACAAGG**GTAAGTGG* *
	OEB12	**GAAGAGCACGTTCTGCGTGATGT(180bp)GCAGCTACAAGG**GTGAGTGG* *
*tim*	ONM	TTTATTTA------------AGTATTAT(45bp)GAGGATATATCTAATC************ *
		TTTATTTATAAATTATCTTATTTATTAT(45bp)GAGGATATCTCCAATC************ *
	OEB12	TTTATTTATAAATTATCTTATYWATTAT(45bp)GAGGATATATCYAATC<1/emph>************ *
		TTTATTTATAAATTATCTTATYWATTAT(45bp)GAGGATATTTCYAATC************ *
*Clk*	ONM	TATATGCCGTTCGAGGTGCTAGGCACCTCTGGTTATGATTACTATCACTT*
	OEB12	TATATGCCGTTYGAGGTGCTGGGCACYTCYGGYTAYGATTACTATCACTT*
*cyc*	ONM	GAGCTTAAGATG(32bp)CGTGGTAGGTTGT(146bp)GTGCCCTAGGGA* * *
	OEB12	GAGCTCAAGATG(32bp)CGTGGTGGGTTGT(146bp)GTGCCCCAGGGA* * *
*cry*	ONM	AGAGGTAACAGGTTCAGTTACGGYTGTATTGCTTTAGTTTTAACTTTWTT************ *
	OEB12	AGAGGTAACAGGTTCAGTTACGGCTGTACTGCTTTAGTTTTAACTTTATT************ *
		AGAGGTAAC------------GGTTGTATTGTTTTAGTTTTAACTTTATT************ *

Asterisks indicate SNPs/deletions used as strain-specific markers. Boldface characters show coding regions equivalent to the *D. melanogaster* sequences used for comparison. Two types of sequences were found in ONM and OEB12 in the case of *tim*, and in OEB12 in the case of *cry*.

### Genotyping with the five circadian clock genes

Dominance of the diapause character in strain ONM was already known from the diapause incidence in F_1_ females (Fig. 1A, B). Therefore, we examined the effect of the circadian genes by backcrossing F_1_ females with OEB12 males (i.e., of the non-diapause strain) and genotyping the BC females. If a gene affected the diapause phenotype, then its frequency of occurrence in females homozygous for the OEB12 alleles would be expected to be low, whereas that in females heterozygous for ONM/OEB12 alleles would be expected to be high.

We genotyped 329 BC females in total: 116 BC females from the backcross using F_1_(ONM×OEB12) females, and 213 BC females from the backcross of F_1_(OEB12×ONM) females. The frequencies of occurrence of the circadian clock genes in females homozygous for the OEB12 allele were not significantly different from those in females heterozygous for the ONM and OEB12 alleles, except in the case of *cyc* (χ^2^  =  7.30, *P*  =  0.007) (Table 3). The relative positions of the genes, as expected from the recombination values (Table 4), were similar to those in *D*. *melanogaster*, which are *per* on X, *tim* on 2L, *Clk* and *cyc* on 3L, and *cry* on 3R (FlyBase, http://flybase.org/).

**Table 3:** Segregation ratio for each gene.

	Frequency			
Gene	Homo	Hetero	χ^2^	*P*
*per*	154	175	1.340	0.247
*tim*	150	179	2.556	0.110
*Clk*	165	164	0.003	0.956
*cyc*	140	189	7.298	0.007
*cry*	182	147	3.723	0.054

“Frequency” shows the numbers of flies homozygous and heterozygous for each gene. A χ^2^-test was conducted under the expectation of a 1:1 segregation ratio between homozygous and heterozygous flies.

**Table 4:** Recombination values between genes.

	Recombination values (%)			
	*tim*	*Clk*	*cyc*	*cry*
*per*	58.97	50.15	48.63	51.06
*tim*		55.62	54.10	55.32
*Clk*			23.40	31.91
*cyc*				40.12

### Association between circadian clock genes and diapause incidence

In our statistical analysis we compared the AICs of all possible combinations of factors (i.e., the five genes) to find the best-fit model. The model that included “diapause  =  *tim*+*cyc*+*cry*” had the smallest AIC (390.2) (Table 5). In this model, *tim* and *cry* were significant (*P*<<0.0001), but *cyc* was not significant (*P*  =  0.079). The AICs of models including and excluding *cyc* were similar: the AIC for the model “diapause  =  *tim*+*cry*” was 391.3. Other models that included other interactions and main factors did not give AICs smaller than 390.2; that is, none of the interactions or the two main factors *per* and *Clk* had any effect on diapause incidence.

**Figure 3:**
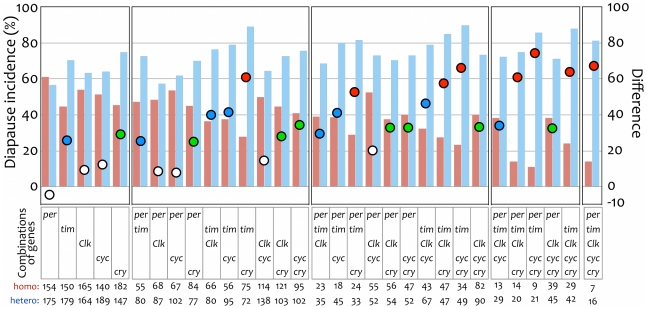
Diapause incidences for each genotype of BC progeny of *Drosophila triauraria* under short daylength conditions, and their differences between heterozygous and homozygous females. Red and blue bars show diapause incidences (%) of flies homozygous and heterozygous for the genes shown as “Combinations of genes” in the middle of the figure, respectively. Numbers of dissected flies are shown at the bottom of the figure for homozygous and heterozygous, respectively. Plotted circles show differences in diapause incidences between heterozygous and homozygous flies of each gene set. The differences indicate the strength of the genetic effects of each gene set on the phenotype: a gene set with a strong genetic effect will show a higher difference. Blue, differences involving *tim*; green, differences involving *cry*; red, differences involving both *tim* and *cry*; white, differences involving neither *tim* nor *cry*. Scale for diapause incidence is shown on the left side, and that for difference is on the right side.

We summarized the diapause incidences in flies of each genotype in Figure 3. Here, the rates of homozygous and heterozygous flies for each gene set are shown as red and blue bar graphs, respectively. Differences between homozygous and heterozygous flies are plotted as circles; the difference values were calculated as “diapause incidence in heterozygous females – diapause incidence in homozygous females” for each gene set. Stronger genetic effects on the phenotype should result in greater differences between flies that carry homozygous alleles vs. those that carry heterozygous alleles. As found above, *tim* and *cry* were highly associated with diapause occurrence.

### Eclosion rhythm

**Figure 4:**
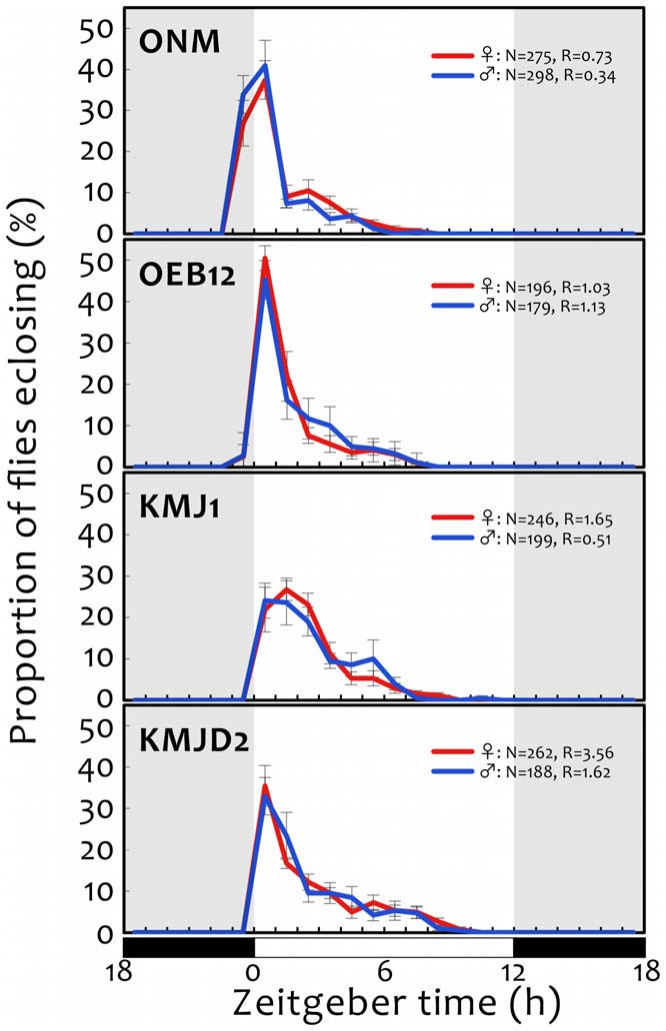
Time-series proportions of *Drosophila triauraria* flies eclosing under 12-h light:12-h dark cycles at 23°C. Eclosing flies were counted every hour over several days. Counted numbers of flies were pooled and the proportions calculated for each data point. Error bars represent ± SEM. R numbers are Winfree's R values.

We determined the eclosion rhythms of 1843 flies (275 females and 298 males from ONM, 196 females and 179 males from OEB12, 246 females and 199 males from KMJ1, and 262 females and 188 males from KMJD2) (Fig. 4). The Winfree's R-values were 0.73 for females and 0.34 for males from ONM; 1.03 for females and 1.13 for males from OEB12; 1.65 for females and 0.51 for males from KMJ1; and 3.56 for females and 1.62 for males from KMJD2. These results show that the eclosion patterns were strongly rhythmic. Furthermore, the phases of the rhythmic patterns clearly differed between diapause and non-diapause strains. About 30% of flies of the diapause strain ONM had eclosed within 1 h before the onset of photophase, and about 40% had eclosed within 1 h after that. In contrast, all three non-diapause strains had barely eclosed before the onset of photophase (2.6% of females and 2.8% of males from OEB12 and 0% of both sexes from KMJ1 and KMJD2).

## Discussion

Using diapause and non-diapause strains, we studied the association between five circadian clock genes and the diapause occurrence, that is photoperiodic response, in *D. triauraria*. Because all four strains that we used had clear eclosion rhythms, we considered that their circadian clocks were functioning normally. Clear differences were found in diapause incidence under SD conditions between the strain from a high-latitude location (ONM, the diapause strain) and the strains from low-latitude locations (OEB12, KMJ1, and KMJD2, the non-diapause strains). Our genotyping analysis revealed that molecular markers within the two genes *tim* and *cry* were associated with this difference in diapause incidence, specifically under SD conditions (not under LD conditions); the three genes (*per*, *Clk*, and *cyc*) had no significant effects.

The involvement of circadian clock genes in diapause induction has been discussed for a long time [4], [11], [19], [47], [48]. Recently, some circadian clock genes have been suggested to be associated with diapause in insects [40], [49]–[56]. Studies using putative mutant strains [49]–[51] or the natural variations in diapause incidence that are often observed with latitudinal clines [40], [52] have shown that *tim* is the gene most likely associated with diapause occurrence. The different isoforms encoded by *tim* alleles associated with diapause occurrence in *D*. *melanogaster* differ in their interactions with the CRY protein, and their circadian photoresponsiveness are different [57]; this seem to be suggestive, even though their findings do not explain the differences in diapause incidence. Here, we found that the occurrence of diapause was associated with the presence of molecular markers in *tim* and *cry*. The proteins encoded by these two genes are the first components in the photoresponse of the circadian clock: CRY is a circadian photoreceptor that triggers TIM degradation in response to light [35]. Involvement of CRY in diapause occurrence has also been suggested in the flesh fly *Sarcophaga similis* [58].

*per* has also been examined in some other insect species, and both positive [53]–[55], [59] and negative [60], [61] results for involvement in diapause have been reported. As these cases, the involvement of *per* was likely partial or depended on the species. Our study clearly showed no association of the *per* locus with diapause in *D. triauraria*, even though at least one locus on the X chromosome is expected to be responsible for diapause in this species [38]. We in fact detected a difference in diapause incidence that seemed to indicate an X-chromosome effect. This difference was observed between two BC females obtained from backcrosses using OEB12 females and hybrid F_1_ males (χ^2^  =  5.5152, *df*  =  1, *P*  =  0.0189; Fig. 1C). In the crosses no recombination was expected, because we had used hybrid male flies. The BC females therefore had different X chromosome pairs: the BC females from the cross with F_1_(OEB12×ONM) males had two X chromosomes from OEB12, and the BC females from the cross with F_1_(ONM×OEB12) males had a pair of X chromosomes from ONM and OEB12.

There had been little study of the involvement of *Clk* and *cyc* in diapause until recently [53], when control of the occurrence of diapause by *cyc* was reported in the bean bug *Riptortus pedestris* by using RNAi [54], [55]. However, we found no association of *Clk* and *cyc* with the diapause occurrence, even though a marginal effect was detected for *cyc* (*P*  =  0.079, Table 5).

**Table 5:** The final model selected as the generalized linear model.

Source	Estimate	SE	*z* value	*P* (>|z|)
(Intercept)	−1.27	0.27	−4.67	<<0.0001
*tim*	1.44	0.26	5.47	<<0.0001
*cyc*	0.45	0.26	1.76	0.079
*cry*	1.48	0.27	5.48	<<0.0001

glm (formula = Diapause ∼ tim+cyc+cry, family = binomial). Null deviance: 446.17 on 328 degrees of freedom. Residual deviance: 382.20 on 325 degrees of freedom. AIC: 390.2.

**Figure 5:**
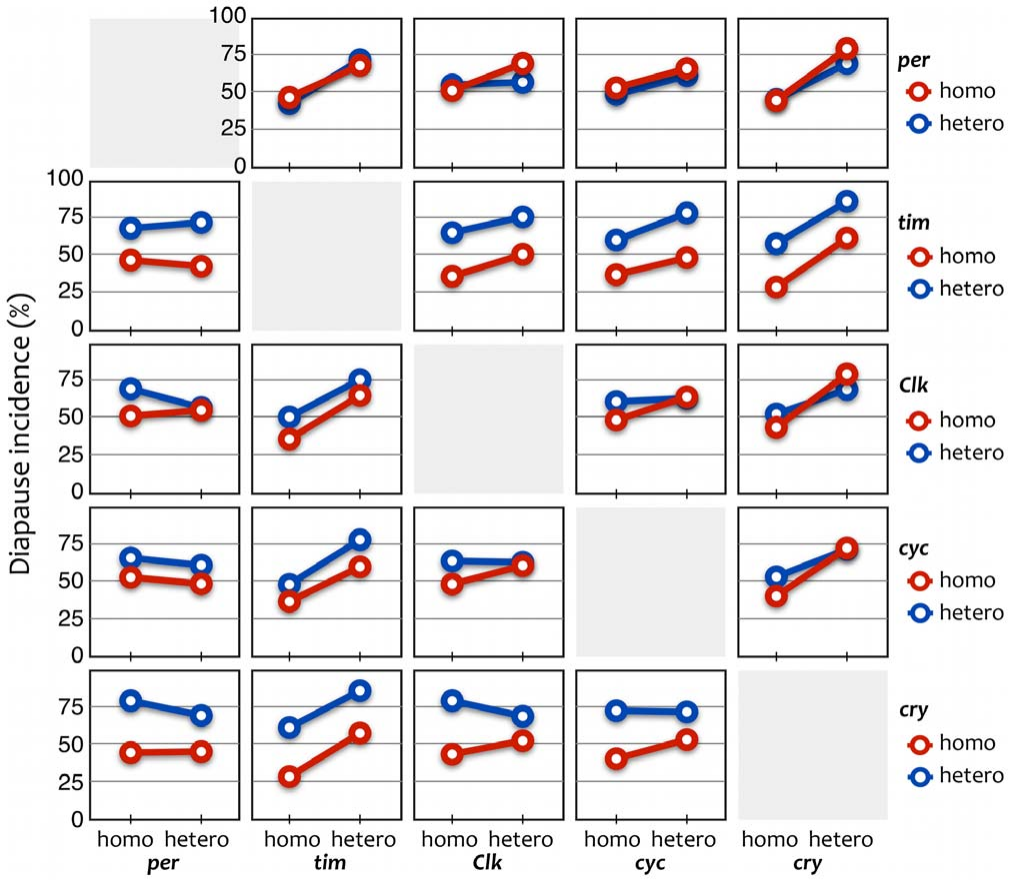
Diapause incidences in allelic combinations of two of the five genes of BC females of *Drosophila triauraria*. “homo,” homozygous for alleles from the non-diapause strain; “hetero,” heterozygous for those from the diapause and non-diapause strains. In the cases of *per*, *Clk*, and *cyc* (rows 1, 3, and 4), the blue and red lines almost overlap in each graph, indicating that there was no allelic effect on diapause incidence. In the cases of *tim* and *cry* (rows 2 and 5), the blue lines are always higher than the red lines in each graph, indicating that there is an effect of allelic differences on diapause incidence; furthermore, the blue and red lines are completely parallel in the combination of *tim* and *cry*, indicating that there is no interaction effect between them. These effects were confirmed by the statistical analysis.

To our knowledge, no one has performed a genetic linkage analysis between the genes responsible for diapause and multiple circadian clock genes in one species at the same time. As with the circadian clock, the photoperiodic clock mechanism can be made up of the interactions of several genes. Taking into consideration these interactions among genes, we can expect genetic linkage analysis with multiple genes to supply useful information. We performed an examination with the five genes simultaneously and then analyzed the interactions between them. We detected no clear interactions in our statistical analysis, even between *tim* and *cry*; this lack of interaction is indicated by the parallel nature of the blue and red lines in Figure 5. Instead, an additive effect of *tim* and *cry* was observed (Fig. 3): the red circles (involvement of both of *tim* and *cry*) showed large differences in diapause incidence between heterozygous and homozygous flies; the blue (*tim*) and green (*cry*) showed intermediate differences; and the white (neither *tim* nor *cry*) had distinctly weak differences. This additivity is also indicated in Figure 5 as separated parallel blue and red lines. These findings suggest that *tim* and *cry* have independent effects on the occurrence of diapause, unlike their action in the circadian clock. The occurrence of diapause might not be based on circadian clock function; alternatively, even if it is based on clock function, natural variations in the photoperiodic response of diapause might depend simply on variations within one or two genes. It seems reasonable for some of the circadian clock genes to be involved in the occurrence of diapause but for the circadian clock function itself not to be involved, as suggested in previous studies [40], [41]. However, the involvement of *cry* suggests that the photoresponse function is common to both clocks.

The dominance of the diapause character in the diapause strain is apparent from the results in F_1_ females (Fig. 1B). The finding of a diapause incidence of about 50% in BC females suggests that the occurrence of diapause is controlled by a single locus. However, this is unlikely, because our genotyping analysis using molecular markers indicated that there were additive effects of multiple loci (Fig. 3), or, at least, two loci located in the regions including *tim* and *cry* and another locus on the X chromosome (but not *per*). The difference in diapause incidences between BC females homozygous for all five genes and those heterozygous for all five was 67.0% (see a red circle on the far-right column in Fig. 3). It was 60.9% between BC females homozygous for both *tim* and *cry* and heterozygous for both (the alleles of the other three genes were not fixed) (see a red circle on the 12th column from the left in Fig. 3), indicating that the incidence of diapause was strongly influenced by the two regions including *tim* and *cry*. However, note that 28.0% of BC females entered the diapause state even if they had *tim* and *cry* alleles from the non-diapause strain (see a red bar on the 12th column from the left in Fig. 3), indicating that there are other genes or regions that control diapause, besides the two regions including the two markers. This is consistent with the findings of a previous study that the differences in photoperiodic response in *D. triauraria* are due to genes at three or four loci, at least one of which is on the X chromosome [38]. Currently, however, we cannot exclude the possibility that the strong effects we detected are caused by genes located near *tim* and *cry*, not by *tim* and *cry* themselves.

Recently, two independent QTL mapping studies revealed that single genes control natural variations in the incidence of diapause in *D. melanogaster*: one is *Dp110*, which encodes insulin-regulated phosphatidylinositol 3-kinase [62], and the other is *couch potato* (*cpo*), which encodes an RNA-binding protein [63]. In the process leading to photoperiodic diapause, at least two components must be considered: one by which photoperiodic information is received and time is calculated, and the other, endogenous, process by which clock information is transmitted to responsive organs, such as the ovaries in reproductive diapause in *Drosophila* [55], [64]. The two abovementioned genes are considered responsible for the latter endogenous process, but not for the former one [29], [62]–[69]. It is not known whether *tim* and *cry* are associated with the photoperiodic timer or the endogenous process. We are interested in whether allelic differences in *Dp110* and *cpo* are associated with differences in the incidence of diapause between *D. triauraria* strains. Both genes are located close to *cry* in *D*. *melanogaster* (*cpo* 90D1-E1, *cry* 91F11, *Dp110* 92F3).

Our results have provided us with clues as to what needs to be investigated next. We are currently establishing recombinant inbred lines between diapause and non-diapause strains of *D. triauraria*. Use of these recombinant inbred lines will enable us to perform fine mapping and to study the functions of *tim* and *cry* in the photoperiodic response, as well as the interactions between these two genes.
